# Sodium selenite attenuates inflammatory response and oxidative stress injury by regulating the Nrf2/ARE pathway in contrast-induced acute kidney injury in rats

**DOI:** 10.1186/s12882-024-03657-0

**Published:** 2024-07-15

**Authors:** Haiyan Xiang, Qianlin Tan, Yun Zhang, Yan Wu, Yaling Xu, Yuanhao Hong, Gen Li

**Affiliations:** 1 Department of Nephrology, Wuhan City Sixth Hospital, Affiliated Hospital of Jianghan University, No. 168 Xianggang Road, Jiang’an District, Wuhan, Hubei 430014 China; 2 Department of Nephrology, Minda Hospital of Hubei Minzu University, Enshi, 445000 China

**Keywords:** Acute kidney injury, Contrast agent, Sodium selenite, Inflammation, Oxidative stress

## Abstract

**Background:**

Contrast-induced acute kidney injury (CI-AKI) is an acute renal complication that occurs after intravascular contrast agent administration. Sodium selenite (SS) is an inorganic source of Se and has potent antioxidant properties. This study intends to examine its anti-inflammatory and antioxidant effects in CI-AKI.

**Methods:**

A rat CI-AKI model was established with the pretreatment of SS (0.35 mg/kg). Hematoxylin-eosin staining was employed for histopathological analysis of rat kidney specimens. Biochemical analysis was conducted for renal function detection. Tissue levels of oxidative stress-related markers were estimated. Reverse transcription-quantitative polymerase chain reaction revealed the mRNA levels of proinflammatory cytokines. Western blotting showed the Nrf2 signaling-related protein expression in the rat kidney.

**Results:**

SS administration alleviated the renal pathological changes and reduced the serum levels of serum creatinine, blood urea nitrogen, neutrophil gelatinase-associated lipocalin, cystatin C, and urinary level of kidney injury molecule-1 in CI-AKI rats. SS attenuated oxidative stress and inflammatory response in CI-AKI rat kidney tissues. SS activated the Nrf2 signaling transduction in the renal tissues of rats with CI-AKI.

**Conclusion:**

SS ameliorates CI-AKI in rats by reducing oxidative stress and inflammation via the Nrf2 signaling.

**Supplementary Information:**

The online version contains supplementary material available at 10.1186/s12882-024-03657-0.

## Introduction

Contrast-induced acute kidney injury (CI-AKI) is an acute renal complication that occurs after the intravascular administration of contrast agents in clinical diagnostic and interventional procedures [1]. The incidence of CI-AKI varies from 3.3 to 14.5% and can reach up to 20% in high-risk patients [2]. Considerable efforts have been made to reduce the incidence of CI-AKI including pretreatment with antioxidants and optimizing hydration protocols, however, it remains the third most prevalent form of hospital-acquired AKI [3]. Hence, it is imperative to develop new strategies to improve the treatment of CI-AKI.

Although the exact mechanisms by which contrast agents induce AKI remain poorly understood, evidence suggests that the direct nephrotoxicity of contrast agents, oxidative stress, and inflammation play pivotal roles in CI-AKI progression [4]. Contrast agents can facilitate the overproduction of reactive oxygen species (ROS) or decrease the activities of antioxidant enzymes, consequently leading to an enhanced oxidative stress environment that impairs renal function [5]. Additionally, continuous oxidative stress leads to the production of inflammatory cytokines, such as tumor necrosis factor (TNF)-α and interleukin (IL)-1β, which result in vascular congestion and interstitial inflammation [6]. Nuclear factor erythroid-derived 2-related factor 2 (Nrf2) is a transcription factor that is critical for maintaining the cellular redox balance. Nrf2 binds to the antioxidant response elements (AREs) to upregulate many antioxidant and detoxifying genes, such as heme oxygenase 1 (HO-1) and NAD(P)H quinone dehydrogenase 1 (NQO1) [7]. The Nrf2/ARE signaling pathway is implicated in antioxidant and anti-inflammatory processes and is considered a significant target in several disorders [8]. Importantly, previous research has demonstrated that activation of Nrf2 signaling transduction exhibits a renoprotective role in CI-AKI [9, 10].

Selenium (Se) is an essential trace element for organisms and is required to sustain different physiological functions [11]. Se is incorporated into the catalytic site of antioxidant enzymes, such as glutathione peroxidase (GSH-Px), thereby playing a critical role in the antioxidant defense mechanism [12]. Sodium selenite (Na_2_SeO_3_, SS) is an inorganic source of Se and is widely used in animal diets for Se supplementation. Previous reports have indicated the anticancer property of SS in several malignancies [13, 14]. Li et al. proposed that SS mitigates mercuric chloride-triggered oxidative stress, apoptosis, and inflammation in the brain tissue by mediating several signaling pathways [15]. Moreover, previous evidence suggested that SS treatment protects against lipopolysaccharide-induced kidney damage in a rat model by increasing renal cell proliferation, reducing cell apoptosis, and enhancing the activities of antioxidant enzymes such as superoxide dismutase (SOD) [16]. Nonetheless, the specific function of SS (Se supplementation) in CI-AKI remains unclarified.

In this study, we intended to explore the function of SS in CI-AKI in a rat model. We speculated that SS could attenuate CI-AKI via its antioxidant and anti-inflammatory properties. The potential mechanism was also under investigation.

## Materials and methods

### Chemicals

SS (99% purity) was purchased from Sigma-Aldrich (#214,485, Shanghai, China). Indomethacin (HY-14,397), iohexol (HY-B0594), and NG-nitro-L-arginine methyl ester (L-NAME; HY-18,729 A) were purchased from MedChemExpress (Shanghai, China).

### Animals

Male Sprague-Dawley rats (6–7 weeks, 180–220 g) were obtained from Cavens (Changzhou, China) and kept under standard conditions (50–60% humidity, 23 ± 1℃, 12-h light/dark cycle) with free access to food and water. The study was approved by the Ethics Committee of Wuhan City Sixth Hospital, Affiliated Hospital of Jianghan University. All experimental procedures were conducted as per the Guide for the Care and Use of Laboratory Animals.

### Animal grouping

**Fig. 1:**
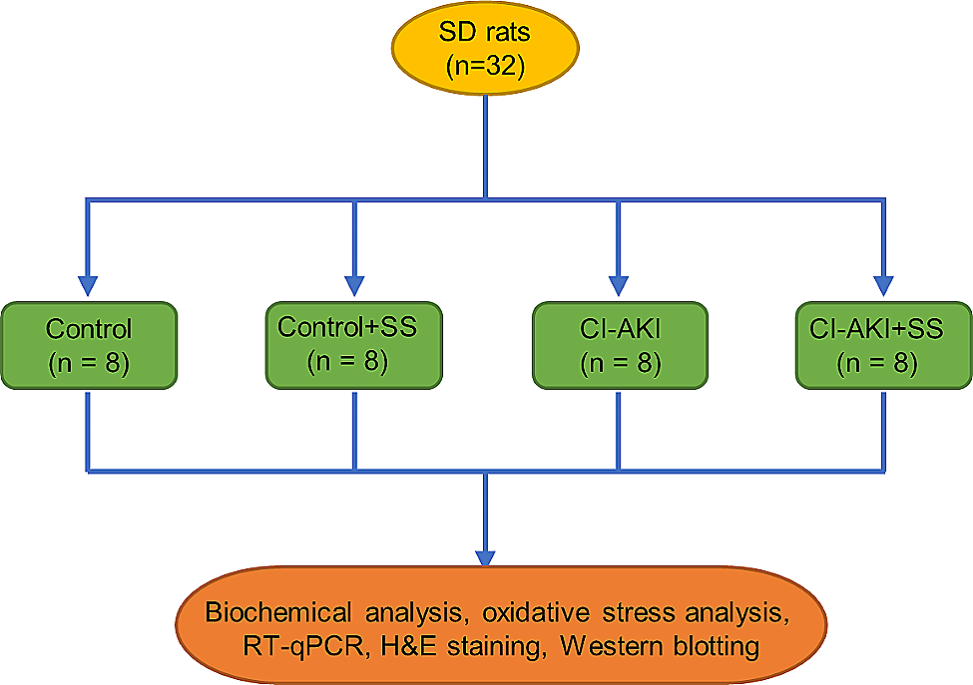
Animal grouping of the study

After one week of acclimatization, 32 SD rats were randomly grouped as: (1) control group; (2) control + SS group; (3) CI-AKI group; and (4) CI-AKI + SS group, with 8 rats per group (shown in Fig. 1). The rat CI-AKI model was established according to previous description [17]. In short, after 12 h of fasting and water deprivation, rats in groups 3) and 4) were injected with 10 mg/kg indomethacin via the tail vein, followed by injection of L-NAME (10 mg/kg) and iohexol (3 g iodine/kg) 15 and 30 min later, respectively. Rats in the control groups received the equal volume of normal saline at the same time point. Rats in control + SS and CI-AKI + SS groups received intragastrical administration of 0.35 mg/kg SS (dissolved in deionized water) 48 h before indomethacin injection. The dose of SS was selected based on previous reports [16, 18]. No rats were sacrificed due to toxicity-related symptoms during the experimental period. Twenty-four hours after iohexol injection, all rats were sacrificed under anesthesia by decapitation. The blood, urine, and bilateral kidneys were collected.

### Biochemical analysis

The blood samples were centrifuged at 3000 rpm at 4℃ for 15 min to separate the serum, which was then stored at -80℃ before use. The serum levels of serum creatinine (SCr; C011-2-1), neutrophil gelatinase-associated lipocalin (NGAL; H392-1-1), blood urea nitrogen (BUN; C013-2-1), cystatin C (Cys-C; H336-1-1) and urinary level of kidney injury molecule-1 (KIM-1; H436-1-1) were determined using commercially available assay kits (all from Nanjing Jiancheng Bioengineering Institute, Nanjing, China) as per the manufacturer’s protocols.

### Hematoxylin-eosin (H&E) staining

Fresh right kidney tissue was fixed in 4% paraformaldehyde, paraffin-embedded and sectioned (5-µm-thick). The sections were deparaffined, rehydrated and stained with H&E (C0105S, Beyotime, Shanghai, China) following the manufacturer’s instructions. A light microscope (Leica Microsystems) was employed for histological observation of the stained sections. For semi-quantitative analysis of renal tubular injury, 10 high-power nonoverlapping fields were randomly selected to evaluate the histopathological changes including interstitial edema, cast formation, cytoplasmic vacuolar changes, and luminal congestion. The degree of tubular injury was scored by two investigators blinded to the animal grouping following the criteria: 0, no injury; 1, 0–25% injury; 2, 25–50% injury; 3, 50–75% injury; and 4, 75–100% injury.

### Measurement of oxidative stress-related factors

Left kidney tissue was homogenized in phosphate-buffered saline (1/10 w/v) and centrifuged at 3000 rpm for 10 min. The supernatant was harvested to determine the activities of SOD (A001-3-2), myeloperoxidase (MPO, A044-1-1), and GSH-Px (A005-1-2) using commercially available assay kits (Nanjing Jiancheng Bioengineering Institute) as per the instructions.

### Reverse transcription-quantitative polymerase chain reaction (RT-qPCR)

Total RNA isolation from the kidney was achieved using TRIzol reagent (R0016, Beyotime). The iScript cDNA Synthesis Kit (Bio-Rad, Hercules, CA) was employed for cDNA preparation. Real-time qPCR was conducted on a CFX96 Touch Real-Time PCR Detection System (Bio-Rad) using THUNDERBIRD SYBR^®^ qPCR Mix (Toyobo, Japan). The 2^−ΔΔCt^ method was utilized for calculating relative gene expression, with GAPDH as normalization. Table 1 shows the primer sequences.

**Table 1:** Primers used for RT-qPCR.

Gene	Forward (5’-3’)	Reverse (5’-3’)
TNF-α	CTTCTCATTCCTGCTCGTG	TTTGGGAACTTCTCCTCCT
IL-1β	TTCATCTTTGAAGAAGAGCCC	CTGTCTAATGGGAACATCACAC
TGF-β	TTACCTTGGTAACCGGCTG	CTGTATTCCGTCTCCTTGGT
GAPDH	AACTCCCATTCTTCCACCT	TTGTCATACCAGGAAATGAGC

### Western blotting

RIPA lysis buffer (Thermo Scientific) was employed for the tissue protein extraction, and a BCA assay kit (Beyotime) was employed for the quantification. Protein samples were separated by 10% SDS-PAGE, followed by transferring them onto the polyvinylidene fluoride membranes (Thermo Scientific). Next, the membranes were treated with the blocking buffer (Beyotime), cut into corresponding parts according to the molecular weight, and then incubated with primary antibodies (shown in Table 2) overnight at 4℃. After rinsing thrice with TBST, the membranes were incubated with HRP-conjugated secondary antibody (Abcam, Shanghai, China) for 1 h at room temperature. Blots were developed using an ECL detection kit (Thermo Scientific), and ImageJ software was employed for analyzing the band intensities.

**Table 2:** Primary antibodies used in Western blotting

Target	Host species	Cat. No	Dilution
Nrf2	Rabbit	ab313825*	1:500
Keap1	Rabbit	#32,450#	1:500
HO-1	Rabbit	ab189491*	1:2000
NQO1	Rabbit	ab80588*	1:10000
GAPDH	Rabbit	ab181602*	1:10000

*From Abcam, Shanghai, China; #From Signalway Antibody, Nanjing, China

### Statistical analysis

Normal distribution of data was assessed with Kolmogorov-Smirnov test. Data are expressed as the mean ± standard deviation. Difference comparisons were performed by one-way ANOVA followed by Tukey’s *post hoc* analysis using GraphPad Prism software (version 8.0.2; GraphPad, San Diego, CA). *p*˂0.05 depicted statistical significance.

## Results

### SS ameliorates renal pathological damages in CI-AKI rats

**Fig. 2:**
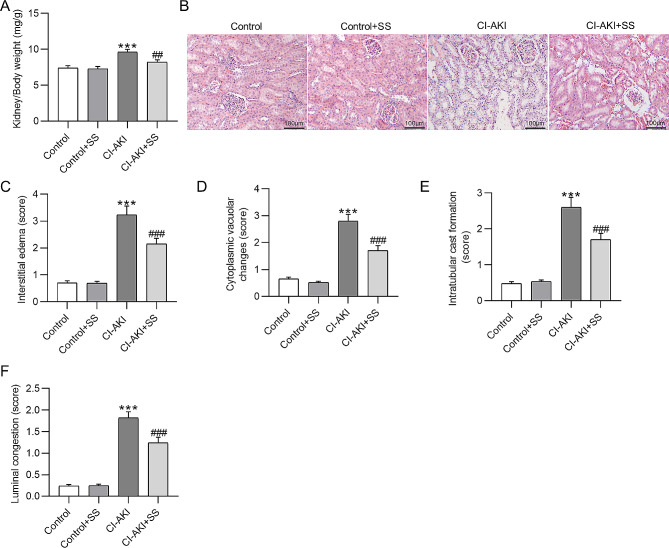
SS ameliorates renal pathological damages in CI-AKI rats. **A**. Measurement of the ratio of kidney weight to body weight in each group. **B**. Representative images of H&E staining for analyzing the morphological changes of rat kidney tissue. **C-F**. Semi-quantitative analysis of interstitial edema, intratubular cast formation, luminal congestion, and cytoplasmic vacuolar changes in each group. ***p˂0.001 vs. control group; ##*p*˂0.01, ###*p*˂0.001 vs. CI-AKI group

We first measured the kidney weight and body weight of rats in each group. As shown in Fig. 2A, the ratio of kidney weight to body weight in CI-AKI rats was markedly increased as compared to that in the control rats (7.4 ± 0.33 vs.9.6 ± 0.41, *p*˂0.001), while SS administration significantly decreased this ratio in CI-AKI rats (9.6 ± 0.41 vs. 8.2 ± 0.32, *p*˂0.01), indicating that SS attenuated renal edema in rats with CI-AKI. H&E staining was conducted to evaluate the morphological changes of the kidney. Relative to the control rats, CI-AKI rats exhibited severe tubular damage. However, pretreatment of SS mitigated contrast agent-induced tubular damage in rat kidney tissue (Fig. 2B). Semi-quantitative results showed that SS significantly alleviated interstitial edema (2.15 ± 0.21 vs. 3.23 ± 0.33, *p*˂0.001), cytoplasmic vacuolar changes (1.71 ± 0.18 vs. 2.8 ± 0.25, *p*˂0.001), intratubular cast formation (1.7 ± 0.18 vs. 2.6 ± 0.28, *p*˂0.001), and luminal congestion (1.24 ± 0.13 vs. 1.82 ± 0.14, *p*˂0.001) in the kidney of rats with CI-AKI (Fig. 2C-F), indicating the protective effect of SS on CI-AKI in rats.

### SS improves renal function in CI-AKI rats

**Fig. 3:**
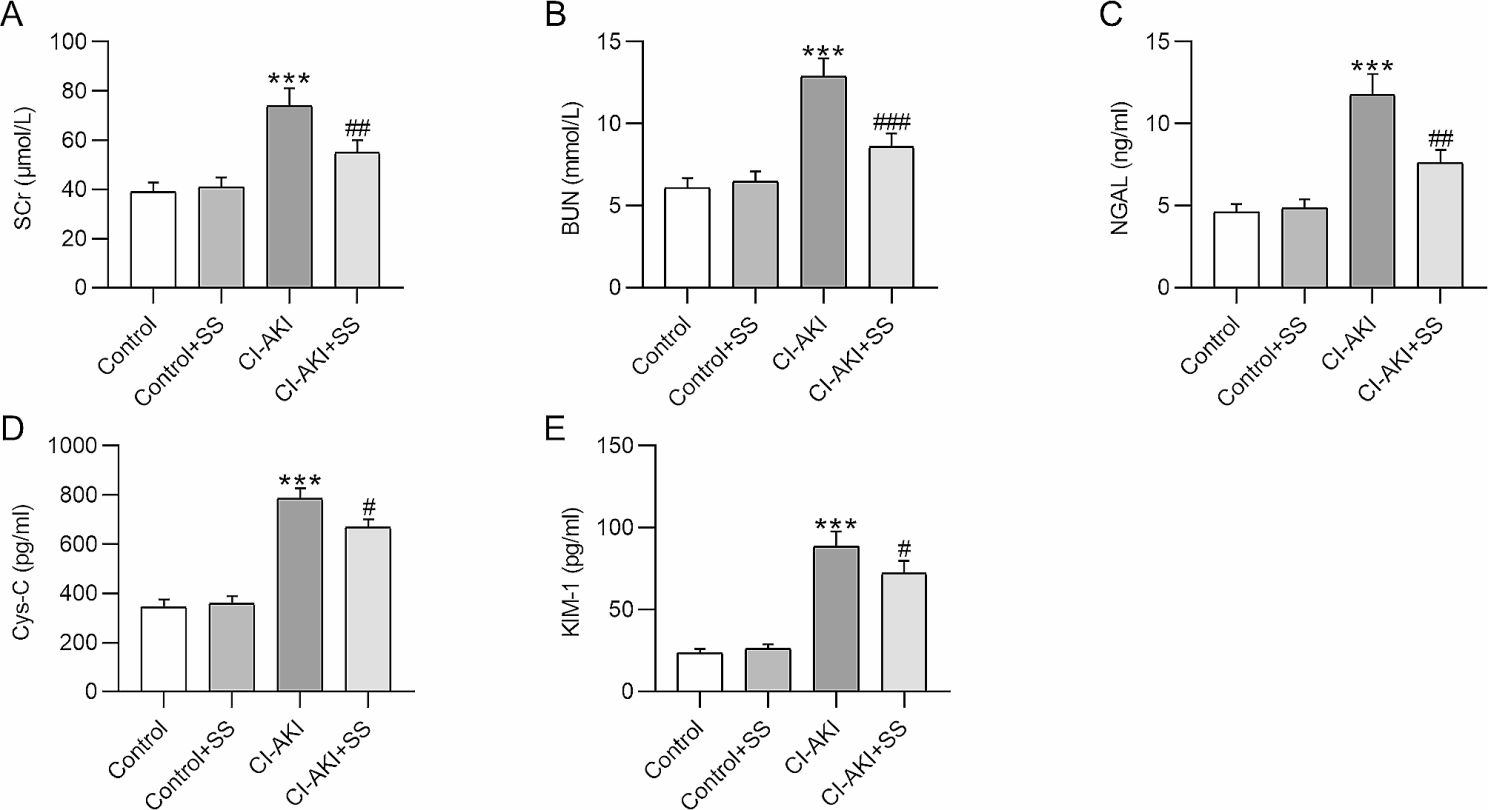
SS improves renal function in CI-AKI rats. **A-E.** Evaluation of serum levels of SCr, BUN, NGAL, Cys-C, and urinary level of KIM-1 in each group. ***p˂0.001 vs. control group; #*p*˂0.05, ##*p*˂0.01, ###*p*˂0.001 vs. CI-AKI group

Biochemical analysis was performed to estimate the SS effect on renal function in rats with CI-AKI. Relative to the control group, the CI-AKI group exhibited markedly elevated serum levels of SCr (39 ± 3.71 vs. 74 ± 7.18, *p*˂0.001), BUN (6.1 ± 0.58 vs. 12.9 ± 1.09, *p*˂0.001), NGAL (4.62 ± 0.48 vs. 11.8 ± 1.24, *p*˂0.001), Cys-C (345.2 ± 30.14 vs. 784.5 ± 42.14, *p*˂0.001) and urinary level of KIM-1 (23.8 ± 26.2 vs. 88.6 ± 9.12, *p*˂0.001) in rats (Fig. 3A-E), confirming the successful induction of renal dysfunction in rats. Moreover, SS administration prominently reduced the concentrations of these markers in CI-AKI rats (Scr: 74 ± 7.18 vs. 55 ± 5.09, *p*˂0.01; BUN: 12.9 ± 1.09 vs. 8.6 ± 0.81, *p*˂0.001; NGAL: 11.8 ± 1.24 vs. 7.63 ± 0.77, *p*˂0.01; Cys-C: 784.5 ± 42.14 vs. 669.1 ± 32.12, *p*˂0.05; KIM-1: 88.6 ± 9.12 vs. 72.4 ± 7.36, *p*˂0.05) (Fig. 3A-E), suggesting that SS could ameliorate renal dysfunction in rats with CI-AKI.

### SS alleviates oxidative stress and inflammation in CI-AKI rats

**Fig. 4:**
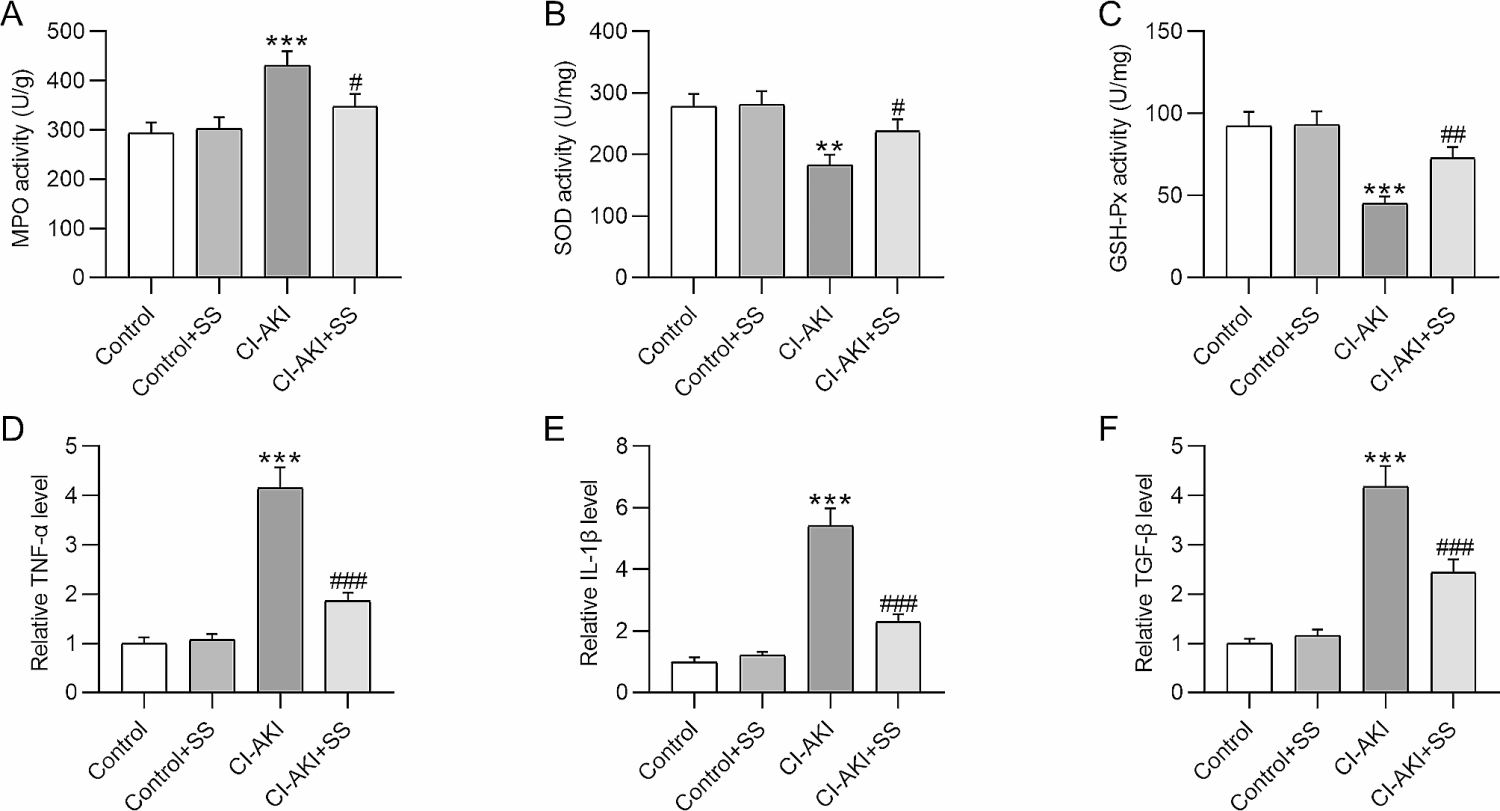
SS alleviates oxidative stress and inflammation in CI-AKI rats. **A-C.** Determination of the activities of MPO, SOA, and GSH-Px in rat kidney tissue. **D-F.** RT-qPCR analysis showing the mRNA levels of TNF-α, IL-1β, and TGF-β in rat kidney samples. **p˂0.01, ***p˂0.001 vs. control group; #*p*˂0.05, ##*p*˂0.01, ###*p*˂0.001 vs. CI-AKI group

Oxidative stress and inflammation are key contributors to the progression of CI-AKI [6]. The results indicated a significant increase in MPO level (293.4 ± 22.15 vs. 431.4 ± 28.52, *p*˂0.001) and a decrease in SOD (278.1 ± 20.41 vs. 183.4 ± 16.14, *p*˂0.01) and GSH-Px (92.3 ± 8.91 vs. 45.3 ± 4.07, *p*˂0.001) activities in the kidney of CI-AKI rats in comparison to the control rats (Fig. 4A-C). Nevertheless, SS administration reduced MPO activity (431.4 ± 28.52 vs. 348.1 ± 25.24, *p*˂0.05) and elevated SOD (183.4 ± 16.14 vs. 238.4 ± 18.74, *p*˂0.05) and GSH-Px (45.3 ± 4.07 vs. 72.8 ± 6.76, *p*˂0.01) activities in the kidney of CI-AKI rats (Fig. 4A-C), suggesting the antioxidant effect of SS on CI-AKI. Furthermore, RT-qPCR analysis depicted that the mRNA levels of proinflammatory factors (TNF-α, IL-1β and TGF-β) in CI-AKI rats were much higher than those in the control rats (TNF-α: 1.00 ± 0.12 vs. 4.15 ± 0.42, *p*˂0.001; IL-1β: 1.00 ± 0.15 vs. 5.42 ± 0.56, *p*˂0.001; TGF-β: 1.00 ± 0.09 vs. 4.18 ± 0.42, *p*˂0.001), which indicated that contrast agent triggered marked inflammatory response in the kidney of rats (Fig. 4D-F). However, pretreatment of SS led to a prominent reduction in the mRNA levels of these proinflammatory factors in the kidney of CI-AKI rats (TNF-α: 4.15 ± 0.42 vs. 1.86 ± 0.17, *p*˂0.001; IL-1β: 5.42 ± 0.56 vs. 2.31 ± 0.24, *p*˂0.001; TGF-β: 4.18 ± 0.42 vs. 2.45 ± 0.25, *p*˂0.001) (Fig. 4D-F). Collectively, SS administration protects against renal oxidative stress and inflammation in rats with CI-AKI.

### SS activates the Nrf2/ARE signaling pathway in CI-AKI rats

**Fig. 5:**
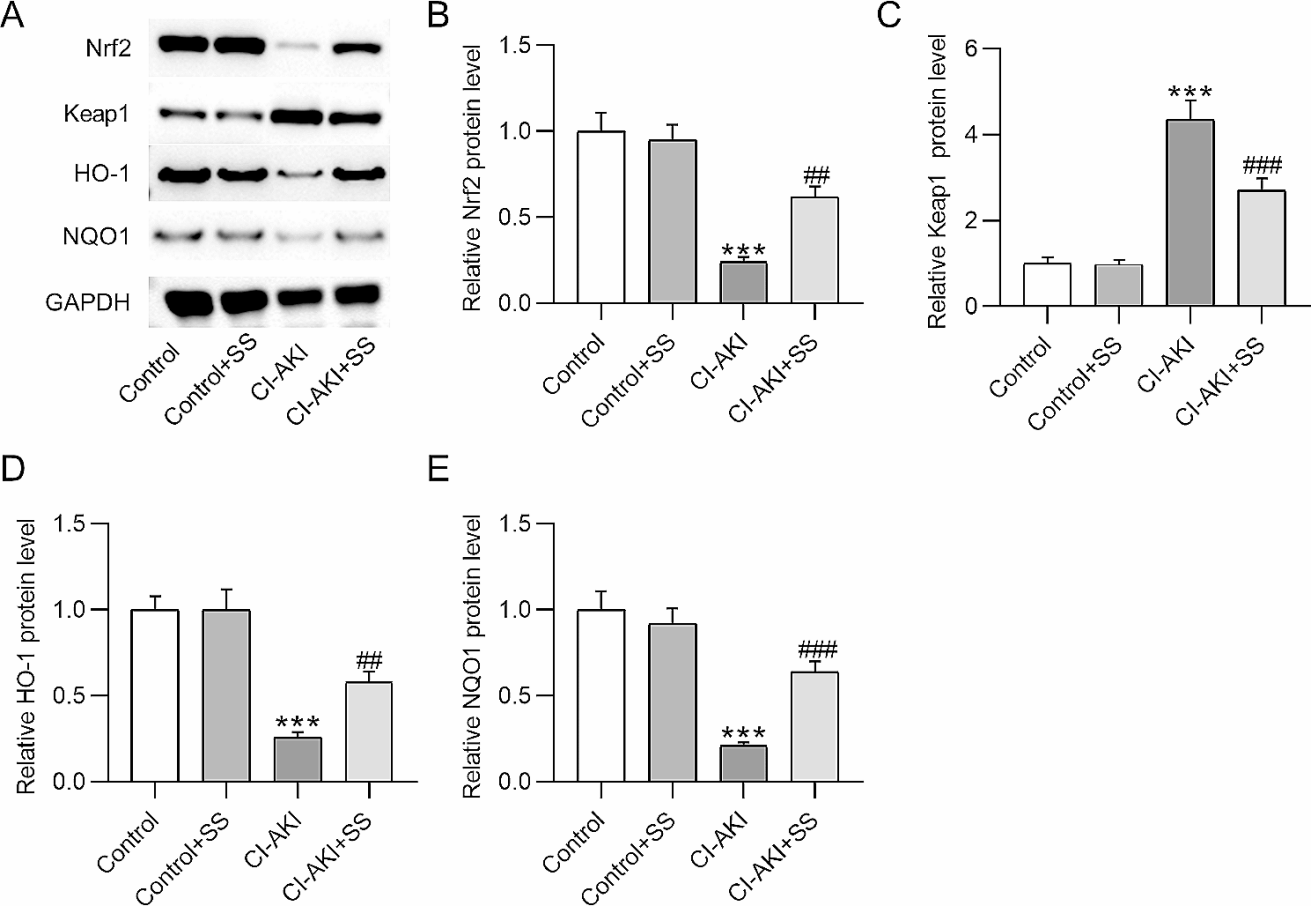
SS activates Nrf2/ARE signaling transduction in CI-AKI rats. **A**. Western blotting showing protein expression of Nrf2, Keap1, HO-1, and NQO1 in rat kidneys. **B-E.** Quantification of protein expression in each group. ***p˂0.001 vs. control group; ##*p*˂0.01, ###*p*˂0.001 vs. CI-AKI group

For exploring SS potential mechanisms in protecting against CI-AKI, we estimated the SS effect on the Nrf2/ARE signaling pathway in CI-AKI rats. As displayed by western blotting, protein expression of Nrf2 (1.00 ± 0.11 vs. 0.24 ± 0.03, *p*˂0.001), its downstream targets HO-1 (1.00 ± 0.08 vs. 0.26 ± 0.03, *p*˂0.001) and NQO1 (1.00 ± 0.11 vs. 0.21 ± 0.02, *p*˂0.001) were prominently downregulated, while Keap1 protein expression was upregulated (1.00 ± 0.15 vs. 4.36 ± 0.44, *p*˂0.001) in the renal tissue of CI-AKI rats. Nonetheless, the above effects were significantly abated in CI-AKI rats pretreated with SS (Nrf2: 0.24 ± 0.03 vs. 0.62 ± 0.06, *p*˂0.01; Keap1: 4.36 ± 0.44 vs. 2.71 ± 0.28, *p*˂0.001; HO-1: 0.26 ± 0.03 vs. 0.58 ± 0.06, *p*˂0.01; NQO1: 0.21 ± 0.02 vs. 0.64 ± 0.06, *p*˂0.001) (Fig. 5A-E), suggesting that SS promoted the activation of the Nrf2/ARE signaling transduction in the renal tissue of CI-AKI rats.

## Discussion

This study revealed that SS administration could ameliorate renal pathological changes and improve renal function in rats with CI-AKI. Moreover, our results depicted that SS prominently alleviated inflammatory response and oxidative stress damage in the renal tissue of CI-AKI rats, and the mechanism might be associated with the activation of Nrf2/ARE signaling transduction.

Previous evidence has indicated that SS can prevent mercuric chloride-triggered kidney damage in rats via its anti-inflammatory and antioxidant activities [19]. Moreover, it has been reported that SS can protect against lipopolysaccharide-evoked renal injury [16]. Similarly, our study revealed that SS ameliorated CI-AKI in rats, as evidenced by the decreased levels of SCr, BUN, NGAL, Cys-C, and KIM-1 as well as mitigated pathological changes in the kidneys of CI-AKI rats. Evidence has suggested that excessive uptake of SS results in Se poisoning known as selenosis [20]. In addition, SS is rapidly absorbed by plasma and tissues and eliminated slowly after intragastric administration. Thus, in the prevention of selenosis, multiple low-dose Se supplementation is much better than single high-dose Se supplementation [21].

Oxidative stress is considered a critical regulator in CI-AKI pathogenesis, which is caused by either overproduction of ROS or insufficiency of the antioxidant defense system [22]. Contrast agents upregulate the production of ROS and lead to ischemic tubular injury [23]. MPO is a peroxidase enzyme associated with enhanced oxidative stress and inflammation [24]. Many reports have demonstrated the potent antioxidant activity of SS [15, 25]. For example, Ala et al. presented that SS prominently reduces MPO activity in the colon tissues of rats with colitis [25]. Li et al. revealed that SS elevates the levels of several antioxidant enzymes in response to mercuric chloride exposure, including GSH-Px and SOD, thereby mitigating mercuric chloride-evoked oxidative stress in the brain of chickens [15]. Consistent with above reports, our study depicted that SS administration prominently reduced MPO activity but enhanced SOD and GSH-Px activities in the renal tissue of CI-AKI rats. Furthermore, oxidative stress and inflammation are closely associated processes [26]. Contrast application has been demonstrated to promote the release of proinflammatory cytokines, such as IL-1β, TNF-α, and TGF-β, in animal models [27]. In contrast, eliminating inflammation can alleviate renal injury [4]. Previous evidence has demonstrated the anti-inflammatory activity of SS in experimental models [28]. Likewise, the present study displayed that pretreatment of SS led to a significant decrease in the mRNA levels of IL-1β, TNF-α, and TGF-β in CI-AKI rat kidney tissues. These results suggested that SS might exert its renoprotective effect against CI-AKI by attenuating oxidative stress and inflammatory response.

Studies have shown that activation of the Nrf2 signaling attenuates CI-AKI [10, 29]. Under normal conditions, Nrf2 is negatively mediated by Keap1 by inducing Nrf2 degradation and preventing its nuclear translocation. Upon activation, Nrf2 is disassociated from Keap1 and translocates into the nucleus, where it transcriptionally upregulates the antioxidant enzymes [30]. Here, our study depicted that Keap1 protein expression was increased and Nrf2, HO-1, and NQO1 protein levels were elevated in rats with CI-AKI, while these effects were prominently suppressed in CI-AKI rats with SS pretreatment, suggesting that SS activated Nrf2/ARE signaling transduction in the injured renal tissue. A recent study proposed that SS ameliorates fluoride-evoked oxidative renal injury in a rat model by activating Nrf2/HO-1/NQO1 signaling [31], which partially supports our results.

In conclusion, this study reveals for the first time that SS can ameliorate CI-AKI by reducing oxidative stress and inflammation via activation of the Nrf2 signaling transduction. Our results may provide a new direction for improving CI-AKI. Moreover, considering the complexity of mechanisms, further studies focusing on the molecular mechanisms of SS will be important to better understand the pathogenesis of CI-AKI and to elucidate the therapeutic potential of SS.

### Electronic supplementary material

Below is the link to the electronic supplementary material.

Supplementary Material 1

## Data Availability

The datasets used and/or analysed during the current study available from the corresponding author on reasonable request.
