# Supplementary figures and images for: Sodium selenite attenuates inflammatory response and oxidative stress injury by regulating the Nrf2/ARE pathway in contrast-induced acute kidney injury in rats
Source: BMC Nephrol. 2024 Jul 15;25:226. doi: 10.1186/s12882-024-03657-0 (PMC11247789; doi:10.1186/s12882-024-03657-0)

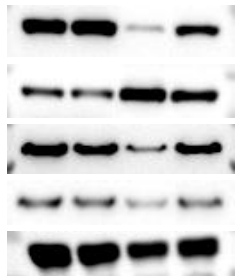

Nrf2 68kDa

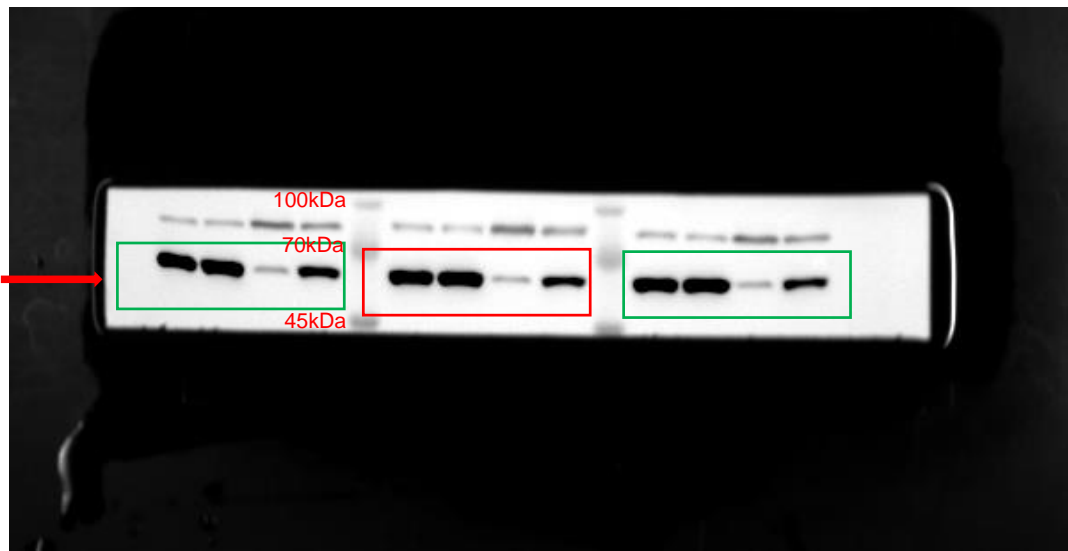

Keap1 70kDa

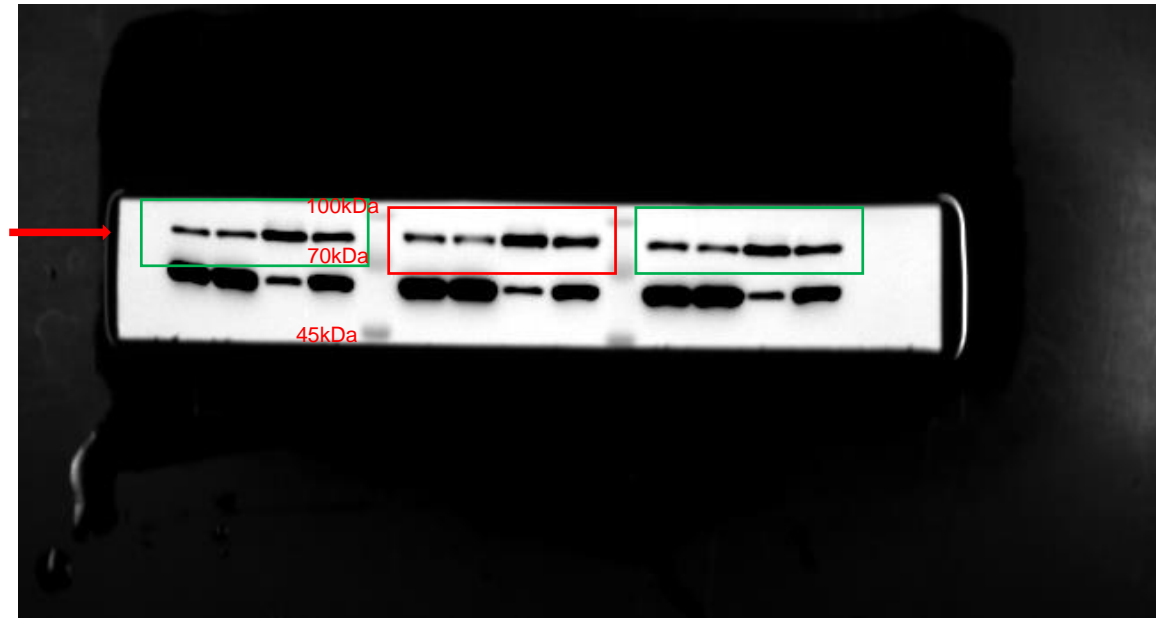

HO-1 32kDa

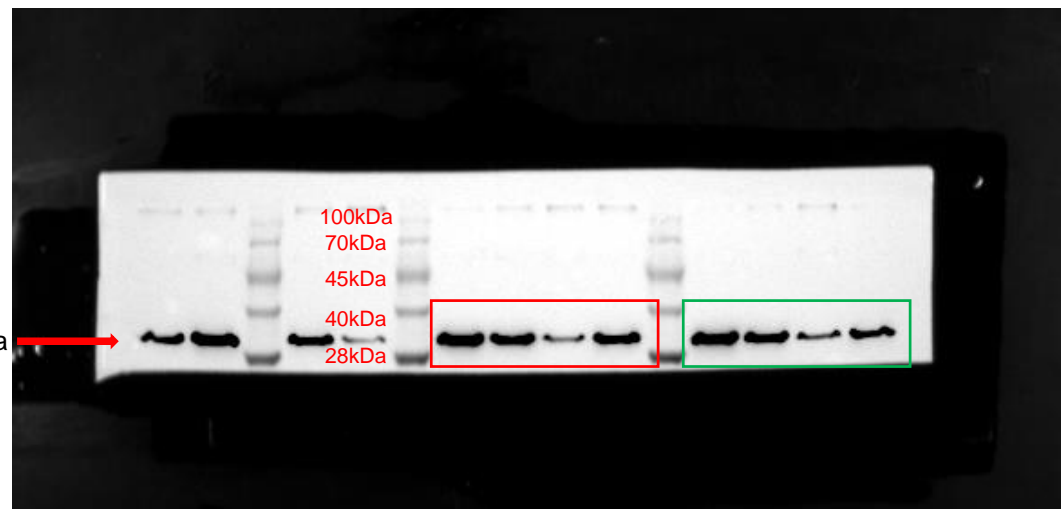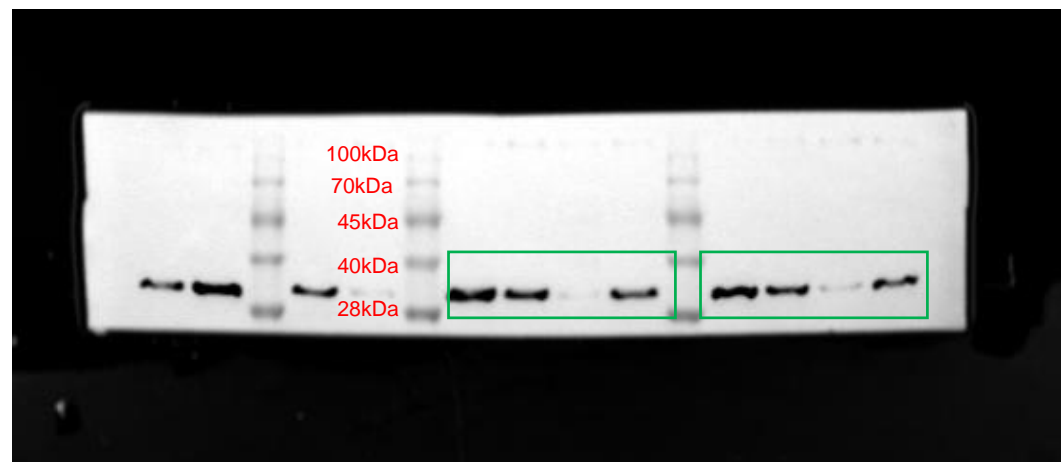

NQO1 31kDa

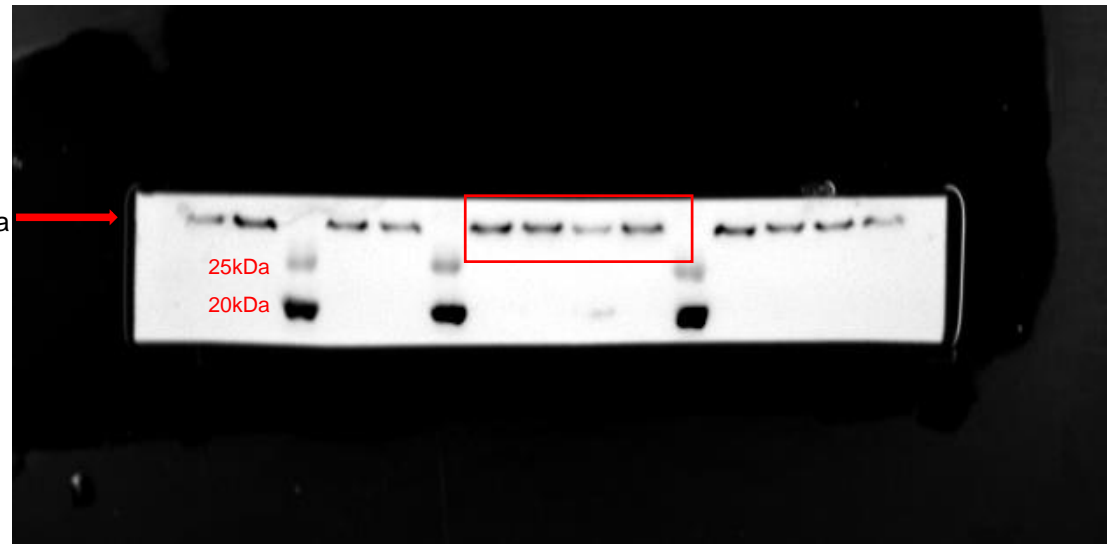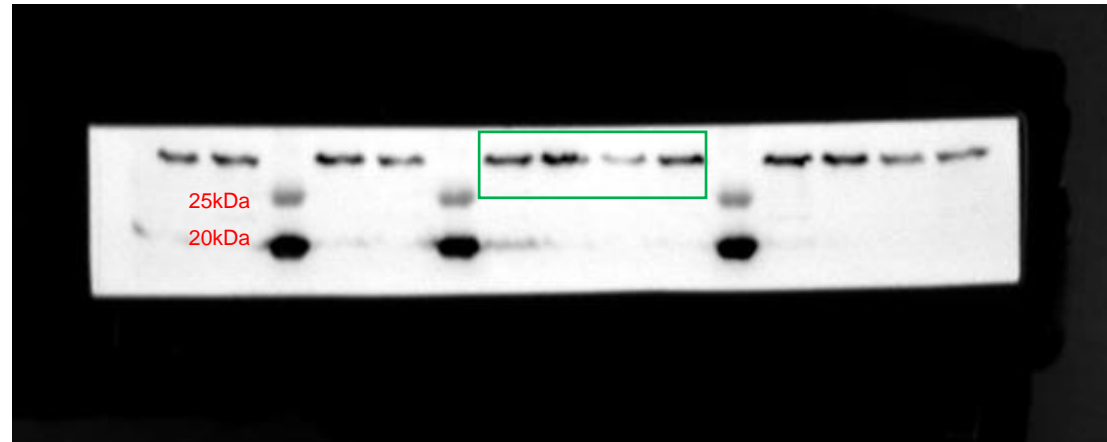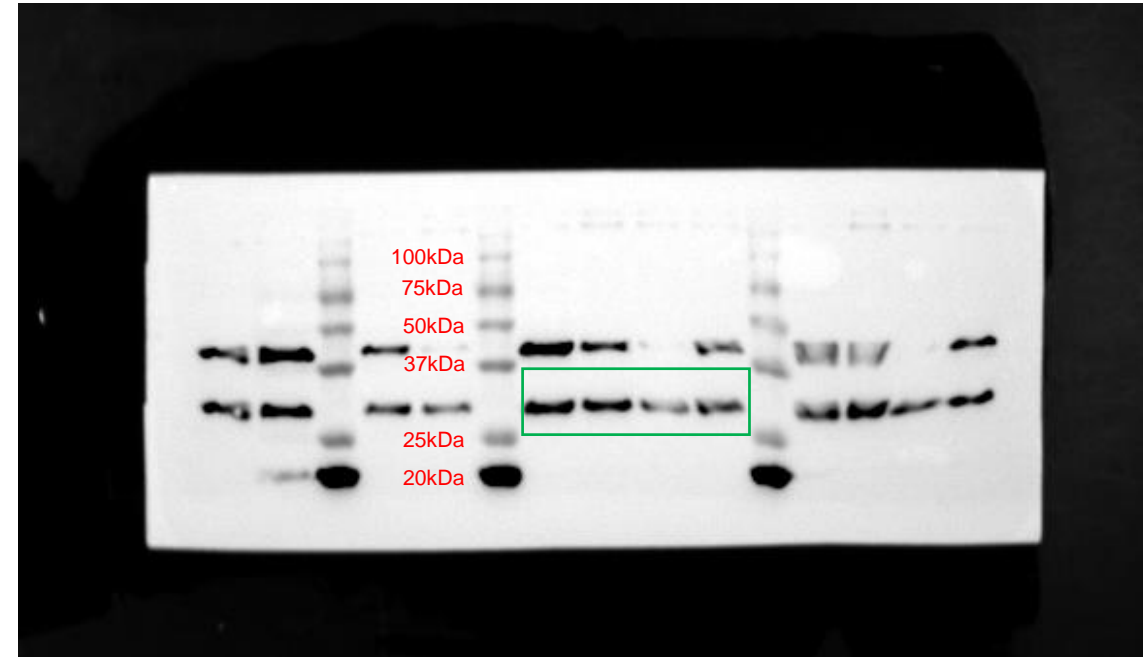

GAPDH 36kDa

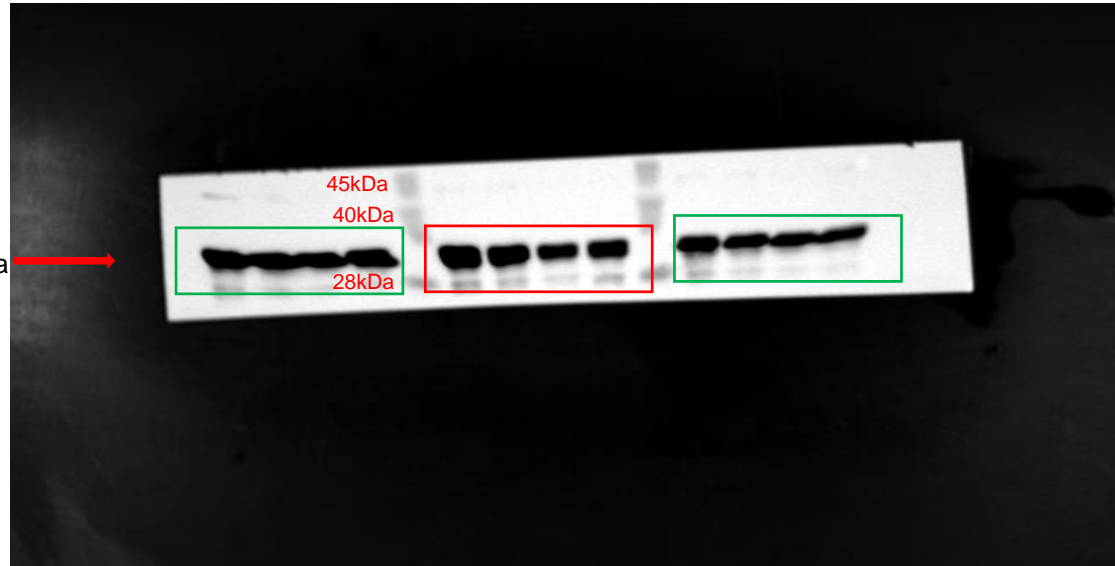

Supplement: Supplementary file 1 — Supplementary Material 1 [file 12882_2024_3657_MOESM1_ESM.pdf]
